# T153. CAN COGNITIVE TRAINING DECREASE REACTIVE AGGRESSION IN SCHIZOPHRENIA?

**DOI:** 10.1093/schbul/sby016.429

**Published:** 2018-04-01

**Authors:** Anthony Ahmed, Matthew Hoptman, Jean-Pierre Lindenmayer

**Affiliations:** 1 Weill Cornell Medical College, NewYork Presbyterian Hospital; 2 Nathan Kline Institute; 3 New York University

## Abstract

**Background:**

Cognitive deficits contribute to aversive social behaviors such as impulsive aggression. Studies have shown that cognitive training interventions may decrease the risk for impulsive aggression. The current study sought to illuminate the underlying mechanism of cognitive training effects on impulsive aggression—particularly, changes in the neural circuitry and in behavioral expressions of emotion regulation and emotion-based impulsivity.

**Methods:**

Participants (N=28) with schizophrenia or schizoaffective disorder were recruited from New York Presbyterian Hospital and Manhattan Psychiatric Center and randomized into one of two cognitive training groups—a cognitive remediation training plus social cognition training (CRT+SCT) group versus CRT alone. At baseline and following 36 hours of training, participants completed the MATRICS Consensus Cognitive Battery (MCCB), Eyes Task, and the Emotion Recognition-40 (ER-40) as measures of neurocognition, mentalizing, and facial affect recognition. We indexed emotion regulation capacity using the Positive and Negative Affect Scale (PANAS) and by obtaining heart rate, respiration, and electrodermal activity while participants viewed pictures selected from the International Affective Picture System (IAPS). A subsample of participants completed fMRI scans during the completion of the emotion regulation task. The Go No-go task and the Emotional Stop Signal task served as measures of impulsivity. Aggression was measured using the Overt Aggression Scale (OAS), the Point Subtraction Aggression Paradigm (PSAP), and the Taylor Aggression Paradigm (TAP).

**Results:**

Participants were 31.93 years old (SD=10.46) and had completed 12.07 (SD=2.59) years of education. Both groups showed improvements from baseline on the composite cognition score of the MCCB with a slight edge to the combined CRT+SCT group (Cohen’s d=0.22). Both groups showed pre-to-post reductions in aggression with only minimal differences. Although both groups showed pre-to-post improvements in affect recognition and mentalizing, the CRT+SCT group showed greater improvements in affect recognition (Cohen’s d = 0.21) and mentalizing (Cohen’s d = 0.39).

Both groups showed reductions in negative affectivity scores from baseline (Cohen’s d =-0.48) but reductions were greater in the CRT+SCT group (Cohen’s d = -0.24). Both groups demonstrated pre-to-post reductions in their Low Frequency/High Frequency heart rate variability ratio (Cohen’s d=-0.83) and pre-to-post reductions in skin conductance (Cohen’s d = -0.48). Pre-to-post differences in HRV and skin conductance were very minimal.

Both groups demonstrated large pre-to-post reductions in misses on the No-Go trials of the Go No-Go Task (Cohen’s d =-1.74). Reductions were greater in the CRT+SCT than the CRT only group (Cohen’s d=0.49) suggesting that the CRT+SCT group show greater improvements in impulse control after cognitive training.

Baseline fMRI scans showed that amygdalofrontal network activation was greater when emotionally evocative pictures were preceded by a reappraisal statement compared to conditions in which they were preceded by negative descriptions. This shows that the emotion regulation task engages relevant neural targets. The presentation will include accumulated follow-up fMRI scans. It is expected that there will be increased BOLD signaling following cognitive training.

**Discussion:**

The study adds to evidence of cognitive training prospects for decreasing aggressive impulses. A mechanistic model with improved emotion regulation and impulse control contributing to reduced aggression may characterize cognitive training effects. Change in neural circuitry of emotion regulation will demonstrate strong proof-of-concept.

